# Comorbidity and Healthcare Expenditure in Women with Osteoporosis Living in the Basque Country (Spain)

**DOI:** 10.1155/2014/205954

**Published:** 2014-10-01

**Authors:** Roberto Nuño-Solinis, Carolina Rodríguez-Pereira, Edurne Alonso-Morán, Juan F. Orueta

**Affiliations:** ^1^O+Berri, Basque Institute for Healthcare Innovation, Torre del BEC (Bilbao Exhibition Centre), Ronda de Azkue 1, 48902 Barakaldo, Spain; ^2^Osakidetza, Basque Health Service, Astrabudua Health Center, Mezo 35, 48950 Erandio, Spain

## Abstract

*Objectives.* This study aimed to establish the prevalence of multimorbidity in women diagnosed with osteoporosis and to report it by deprivation index. The characteristics of comorbidity in osteoporotic women are compared to the general female chronic population, and the impact on healthcare expenditure of this population group is estimated. *Methods.* A cross-sectional analysis that included all Basque Country women aged 45 years and over (*N* = 579,575) was performed. Sociodemographic, diagnostic, and healthcare cost data were extracted from electronic databases for a one-year period. Chronic conditions were identified from their diagnoses and prescriptions. The existence of two or more chronic diseases out of a list of 47 was defined as multimorbidity. *Results.* 9.12% of women presented osteoporosis and 85.04% of them were multimorbid. Although multimorbidity in osteoporosis increased with age and deprivation level, prevalence was higher in the better-off groups. Women with osteoporosis had greater risk of having other musculoskeletal disorders but less risk of having diabetes (RR = 0.65) than chronic patients without osteoporosis. People with poorer socioeconomic status had higher healthcare cost. *Conclusions.* Most women with osteoporosis have multimorbidity. The variety of conditions emphasises the complexity of clinical management in this group and the importance of maintaining a generalist and multidisciplinary approach to their clinical care.

## 1. Background

Multimorbidity, defined as two or more coexisting chronic conditions within an individual [1], is a growing phenomenon in ageing societies and is especially prevalent in older age groups [2–4]. Multimorbidity makes management of chronic conditions by clinicians more complex; they often lack evidence on the best care strategies to follow with this type of patient. In fact, clinical guidelines rarely address multimorbidity and clinical trials often exclude comorbid and older patients [5, 6]. Individuals manifesting multimorbidity are typically associated with higher degrees of disability, lower quality of life, greater psychological distress and mortality risk [7–9], and increased use of health (and social) care [10, 11] services than if we considered these chronic conditions in isolation or individuals with a single chronic condition. It is of particular relevance for patients, their carers, and healthcare providers, but increasingly a concern for policy makers and societies as a whole [7]. Therefore, it is widely accepted that health systems need to focus their strategies in organising healthcare provision and planning for multimorbid patients and pay attention to the implications on treatment patterns and combinations [12, 13].

Because of its worldwide prevalence, osteoporosis is also considered a serious public health concern. Ageing of populations worldwide will be responsible for a major increase in the incidence of osteoporosis in postmenopausal women [14]. The quality of life of postmenopausal women with osteoporosis is adversely affected if they have bone fractures and pain [13].

Previous studies by this research group have revealed high levels (91%) of coexisting disease among women with osteoporosis over 65 years old [15]. Furthermore, osteoporosis and bronchiectasis are the only two diseases, out of a list of 52, disproportionally more prevalent among women living in richer areas in the Basque Country [16].

Osteoporosis is among the most prevalent conditions in the multimorbidity literature. The presence of coexisting conditions in women with osteoporosis has been revealed to reduce health-related quality of life, increase the risk of vertebral fractures, and contribute to mortality [17, 18].

The aim of this study was to establish the prevalence of multimorbidity in women with osteoporosis living in the Basque Country, to categorise the number and types of additional chronic conditions recorded, and to report the demographic and socioeconomic characteristics (age and deprivation index). Finally, healthcare expenditure was estimated for this population.

## 2. Methods

A descriptive study was carried out which included all women aged 45 and above with at least one chronic condition (*N* = 397,940) who were covered by public health insurance in the Basque Country on 31st August 2011 and who had been covered for at least 6 months in the previous year, regardless of whether or not they had made any contact with or use of the Basque Health Service-Osakidetza. The study compared those women with an osteoporosis diagnosis (*N* = 52,844) versus those women without a diagnosis of osteoporosis (*n* = 345,096). The study period was from 1 September 2010 to 31 August 2011. Therefore, we observed almost all of the inhabitants of the Basque Country, by census data in addition to irregular immigrants who have a health identification card and have used the healthcare system during the study period.

Our dataset is derived from the database set up by the population stratification programme (PREST) of Osakidetza. A more detailed description is available by Orueta et al. (2013) [19] and Nuño-Solinís et al. (2012) [20]. In addition, in Osakidetza, diagnoses are coded according to international classification of diseases (ICD-9-MC) [21], while the anatomical, therapeutic, chemical (ATC) [22] coding system is used for drugs prescribed by primary care doctors. With this information, citizens in the Basque Country are classified annually using ACGs (adjusted clinical groups), a case mix system developed at The Johns Hopkins University [23], which enables health problems to be identified from diagnoses and prescriptions, in addition to categorising citizens according to their healthcare needs and cost into a hundred groups.

With the aim of studying multimorbidity and comorbidity of chronic diseases and osteoporosis, we adopted a list of 52 pathologies, defined by consensus among the research team. This task was based on adapting two preexisting lists, published by other authors, the 40 diseases selected by Barnett et al. (2012) [24] and the conditions considered to be chronic in the ACG Technical Reference Guide [23]. A detailed description of this dataset can be found in a previously published article [16].

From the aforementioned list, we omitted four pathologies, “attention deficit disorder,” “intellectual disability,” “anorexia and bulimia” because these diagnoses are very rare in the age group under study, and “prostatic hypertrophy,” because the study includes only women. Therefore, our definitive list was comprised of 47 chronic conditions.

### 2.1. Variables and Analysis

As a social indicator, the deprivation index of census tract was used [25]. A tract is the smallest geographical unit (*n* = 1200 habitants) in which population census data can be broken down; this was created according to population size, geographical, and urban criteria. As the tracts are so small, they tend to be quite homogeneous with respect to the type of dwellings. The deprivation index is an ordinal variable, categorised into five levels, which provides a measure of the socioeconomic characteristics of census tracts and is drawn from the following factors: manual workers, unemployment, temporary employees, and inadequate level of education in the population overall and in young people. The first quintile represents the richest and the fifth quintile the poorest.

We measured health care provision in terms of cost-weighted utilisation of health care. Health care use was estimated for a 12-month period (from 1 September 2010 to 31 August 2011). We consider the cost of the following types of services separately: primary care (including visits to physicians and nurses, laboratory test, and radiology examinations), specialised outpatient care (visits to specialists, rehabilitation, dialysis, radiotherapy, and chemotherapy services), inpatient stays, emergency department attendance, and prescribing. In the case of prescribing, the cost was computed directly from primary care prescriptions recorded in the electronic health records. For other types of use, the number of services for each patient was multiplied by a standardised cost. The costs of hospitalisation and outpatient surgery were calculated in relation to their weight in the corresponding diagnosis related groups (DRGs). Information on some services was not available and these were therefore excluded from the analysis, admission to psychiatric hospitals, home hospitalisation and day care services (except for procedures and services listed above), health care transport, and prostheses and other equipment provided to patients at home.

The prevalence of osteoporosis stratified by age group and deprivation index was obtained; the nonparametric Kruskal-Wallis test was applied to see whether there were differences between these groups. The number of chronic comorbidities for women with osteoporosis was calculated; this was compared against the chronic women without osteoporosis by the nonparametric Wilcoxon Mann-Whitney test. In addition, the average of chronic diseases for women with osteoporosis and without osteoporosis aged >44, stratified by deprivation index, was performed. The 47 risk ratios for the list of chronic conditions with osteoporosis as comorbidity were calculated. Moreover, the average of the observed healthcare costs in women with osteoporosis was obtained.

Statistical calculations were performed using Stata, Data Analysis and Statistical Software, Release 12 (StataCorp, LP, College Station, TX, USA).

## 3. Results

Out of 579,575 women above 44 years, 52,844 (9.12%) presented osteoporosis.  Table 1 shows the prevalence and distribution of women with osteoporosis according to age band and deprivation index. It can be observed that the higher percentage of these is aged 55 to 64 (24.73%) and furthermore the prevalence of this disease increased up to the age of 80. As for the deprivation index we observed that osteoporosis presents more commonly in women with a higher socioeconomic level (24.50%). The prevalence of osteoporosis was higher among rich people. However, no decreasing gradient was observed. After applying the nonparametric Kruskal-Wallis test, statistically significant differences were obtained between the different age bands (*P* < 0.001) and between the different deprivation index groups (*P* < 0.001).

Table 2 shows the number of chronic pathologies for women with and without osteoporosis; the latter subgroup was comprised of 345,096 women. It was observed that only 14.96% of women with osteoporosis only suffer from this chronic disease compared to 36.59% of chronic women over 44 without osteoporosis. It is notable that 1.47% of women with osteoporosis have 10 or more chronic pathologies, compared to 0.35% of women without osteoporosis. The difference in the distribution of the number of chronic pathologies between these two groups was statistically significant (*P* < 0.001). Furthermore, a decreasing gradient was observed in both population subgroups. Therefore, it can be stated that 85.04% of women with osteoporosis and 63.41% of women without osteoporosis over 44 have multimorbidity.

**Figure 1:**
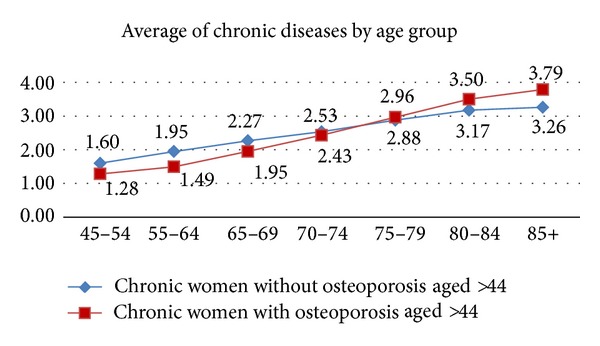
Average of chronic conditions in women with and without osteoporosis by age group.

**Figure 2:**
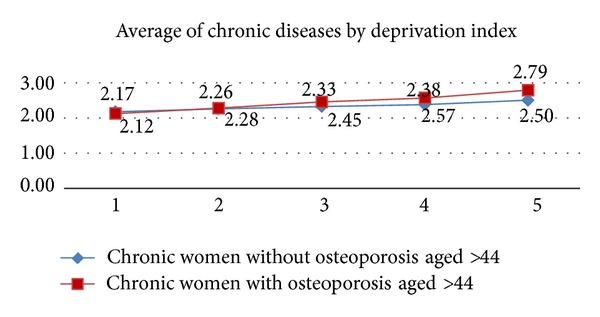
Average of chronic conditions in women with and without osteoporosis by deprivation index.

Figure 1 shows the average of the number of chronic pathologies of women over 44 with and without osteoporosis by age and Figure 2 shows the average of the number of chronic pathologies by deprivation index in the same groups. As can be observed, this average increased with age and socioeconomic index and was higher in more depressed areas. Comparing both groups, the chronic women without osteoporosis have higher chronic condition average until 75 years; after this age women with osteoporosis have higher average. However, from socioeconomic levels 2 to 5, women with osteoporosis have greater chronic condition averages.

The association between osteoporosis and other chronic conditions was statistically significant in 38 of them.  Table 3 shows the risk ratios between osteoporosis and the other chronic pathologies. The people with osteoporosis have nearly two times more risk of suffering bronchiectasis (1.7) than women without osteoporosis. However, women with osteoporosis have less risk of having diabetes (0.65) than women without osteoporosis.

**Figure 3:**
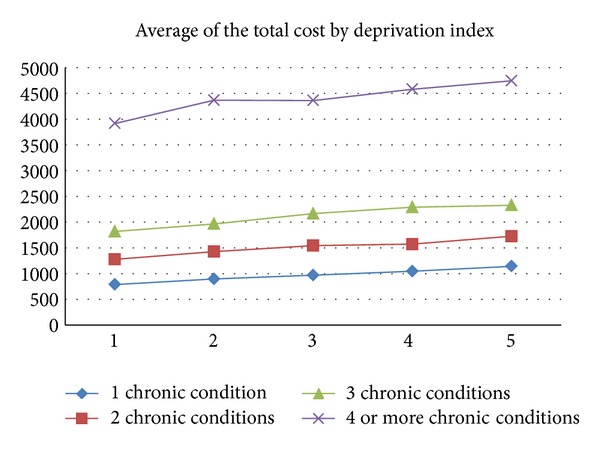
Observed average healthcare cost in women with osteoporosis by number of chronic conditions and deprivation index.

Regarding cost analysis by deprivation index, we checked that, as socioeconomic level reduces, healthcare costs increase in women with osteoporosis both with one pathology and as the number of these pathologies increases (see Figure 3).

## 4. Discussion

This population-based study covering the whole female population over 44 years of a large region analysed the prevalence of multimorbidity in this population and indicated that 85.04% of women in this age group with osteoporosis have multimorbidity. These figures are much higher than those found in other studies performed with a Spanish population in which the prevalence of multimorbidity is approximately 30% [2]. These differences may be because of the difference in methodologies used, which makes comparison of results difficult [1].

In contrast, the prevalence of osteoporosis is less than that found in other studies, also performed on a Spanish population [26]. The results reveal that osteoporosis, when presented in an isolated manner, appears more prevalent, or at least more diagnosed, in people with a high socioeconomic level. However, multimorbidity increases as socioeconomic level decreases. These data coincide with those of other studies that reveal the presence of a relationship between socioeconomic level and multimorbidity [27–30]. The prevalence of osteoporosis increases with age up to 85+ years where it plateaus and declines. This pattern was also seen for most chronic conditions in the Basque Country [15] but another study shows the contrary [31] in the oldest women in Canada. Further analysis is needed.

We have seen that the fact that a woman had osteoporosis increased the risk of having other musculoskeletal disorders.

As socioeconomic level decreases, the health cost is higher. These results coincide with those found in other studies performed in the Basque Country, where we observe that people with a low socioeconomic level use more health resources [16]; that is, inequality in favour of the poor seems to be within Osakidetza. These data could be accounted for by the fact that people with more financial resources use private healthcare to a greater extent than the least affluent to avoid waiting lists [31, 32]. Perhaps the greater use of private healthcare can also account for the higher prevalence of osteoporosis present at higher socioeconomic levels, not because of the actual presence of more osteoporosis in this population but rather because this diagnosis is made more commonly in private healthcare.

A difference between this study and others found in the literature is that the study is performed using data from a health system with universal cover; this includes virtually the entire Basque Country population which reduces the bias that could occur using a restricted population sample. The database used contains primary care, specialised care, and outpatient hospital care information. This use of different data sources reduces the imprecision that could arise in the calculations [33, 34] and leads to a better description of health problems [35].

### 4.1. Limitations of the Study

Given that the administrative databases only contain information on those patients who have requested healthcare, the prevalence obtained only reflects known cases. Those cases, where professionals or patients are unaware, have been excluded; and this is commonplace in the case of osteoporosis. Another limitation is the fact that there is no access to information from private health sector resources. Therefore, there are no data on monitoring of the disease in this sector. Finally, since we use a socioeconomic index collected through area of residence, our study has the limitations of ecological studies.

### 4.2. Practical Implications

Because osteoporosis usually presents together with other chronic diseases, it is important for clinicians to consider the interactions that other multimorbid pathologies and their treatments can have on this. Its onset, frequently insidious, may mean that treatment is focused on another more serious chronic pathology, leaving osteoporosis and its treatment to one side [36]. However, this disease is a significant health problem because of the serious consequences of bone fractures [18]. With the ageing of the population, it is expected that the number of fractures will increase considerably over the next few years along with healthcare costs [14]. Suitable treatment of multimorbidity in people who suffer from osteoporosis, which slows down course and prevents fractures, is a challenge for health systems.

## 5. Conclusions

A high percentage of women with an osteoporosis diagnosis presents at least one other chronic disease and the prevalence of multimorbidity is much higher in women with disadvantaged socioeconomic levels. The comorbidity present in osteoporosis patients should be considered for both correct clinical management and setting up suitable treatments and when drawing up health policies.

## Figures and Tables

**Table 1:** Prevalence of chronic women with osteoporosis by age group and deprivation index.

	*N* females	*N* females with osteoporosis	Prevalence
Age groups			
45–54	176,844	3,197	1.81%
55–64	142,591	13,066	9.16%
65–69	62,315	8,343	13.39%
70–74	48,447	7,400	15.27%
75–79	55,224	8,670	15.70%
80–84	45,364	6,955	15.33%
85+	48,790	5,213	10.68%
Deprivation index			
1	129,894	12,947	9.97%
2	122,902	10,935	8.90%
3	114,130	10,031	8.79%
4	110,174	9,842	8.93%
5	102,475	9,089	8.87%

Total	579,575	52,844	9.12%

*N* represents the population number.

**Table 2:** Distribution of women with osteoporosis and without osteoporosis by the number of comorbidities.

Number of chronic conditions	Number of women without osteoporosis	Percent of women without osteoporosis	Number of women with osteoporosis	Percent of women with osteoporosis
1	126,284	36.59%	7,904	14.96%
2	89,732	26.00%	11,144	21.09%
3	56,847	16.47%	10,933	20.69%
4	33,424	9.69%	8,465	16.02%
5	18,641	5.40%	5,856	11.08%
6	9,776	2.83%	3,592	6.80%
7	5,190	1.50%	2,182	4.13%
8	2,667	0.77%	1,264	2.39%
9	1,321	0.38%	726	1.37%
10 or more	1,214	0.35%	778	1.47%

Total	345,096	100.00%	52,844	100.00%

**Table 3:** Risk ratios for the 47 chronic conditions with osteoporosis as comorbidity.

	Risk ratio	CI at 95%	
		Lower bound	Upper bound
Hypertension	0.81	0.80	0.82
Asthma (currently treated)	1.18	1.14	1.22
Ischemic heart disease	1.10	1.06	1.15
Diabetes mellitus	0.65	0.64	0.67
Hypothyroidism	0.93	0.91	0.95
Rheumatoid arthritis and autoimmune and connective tissue diseases	1.45	1.40	1.50
Deafness and hearing loss	1.13	1.08	1.17
Emphysema, chronic bronchitis, and COPD	1.31	1.26	1.35
Irritable bowel syndrome	1.35	1.26	1.44
Malignancies	1.07	1.04	1.10
Cerebrovascular disease	1.22	1.18	1.26
Chronic kidney disease	1.07	1.02	1.12
Diverticular disease of intestine	1.55	1.49	1.61
Peripheral vascular disease	1.17	1.04	1.31
Heart failure	1.19	1.14	1.25
Glaucoma	1.06	1.03	1.09
Dementia	1.01	0.97	1.04
Schizophrenia, affective psychosis, or bipolar disorder	0.62	0.57	0.68
Inflammatory bowel disease	1.13	1.03	1.24
Parkinson's disease	1.22	1.15	1.29
Multiple sclerosis	0.84	0.69	1.03
Chronic liver or pancreatic disease	1.11	1.05	1.19
Paralysis or muscular dystrophy	1.18	1.10	1.27
Chronic heart disease and others	1.21	1.17	1.26
VIH	0.37	0.24	0.56
Hematologic chronic disorders	1.04	0.93	1.16
Chromosomal anomalies or inherited metabolic disorders	0.98	0.92	1.05
Transplant status	1.16	0.97	1.41
Disorders of the immune system	1.46	1.36	1.57
Degenerative joint disease	1.40	1.38	1.43
Peripheral neuropathy and neuritis	0.86	0.83	0.89
Gout	0.88	0.80	0.97
Treated constipation	1.60	1.51	1.69
Psoriasis or eczema	1.08	0.96	1.21
Migraine	0.82	0.74	0.91
Alcohol problems	0.66	0.56	0.79
Bronchiectasis	1.70	1.59	1.83
Depression	1.12	1.09	1.14
Epilepsy (currently treated)	0.85	0.77	0.95
Atrial fibrillation	1.08	1.05	1.12
Viral hepatitis	1.03	0.94	1.12
Low back pain	1.25	1.22	1.28
Chronic sinusitis	1.10	0.99	1.23
Abuse substances	0.83	0.59	1.16
Treated dyspepsia	1.42	1.39	1.45
Anxiety and other neurotic, stress related, and somatoform disorders	0.86	0.84	0.87
Blindness and low vision	1.07	1.03	1.12

## Abbreviations

ICD-9-MC: International classification of diseases
ATC: Anatomical therapeutic chemical classification system
ACGs: Adjusted clinical groups
DRGs: Diagnosis related groups
ADGs: Aggregated diagnosis groups.
